# Super-Spreader Identification Using Meta-Centrality

**DOI:** 10.1038/srep38994

**Published:** 2016-12-23

**Authors:** Andrea Madotto, Jiming Liu

**Affiliations:** 1 Department of Computer Science, Hong Kong Baptist University, Kowloon Tong, Hong Kong

## Abstract

Super-spreaders are the nodes of a network that can maximize their impacts on other nodes, e.g., in the case of information spreading or virus propagation. Many centrality measures have been proposed to identify such nodes from a given network. However, it has been observed that the identification accuracy based on those measures is not always satisfactory among different types of networks. In addition, the nodes identified by using single centrality are not always placed in the top section, where the super-spreaders are supposed to be, of the ranking generated by simulation. In this paper we take a meta-centrality approach by combining different centrality measures using a modified version of Borda count aggregation method. As a result, we are able to improve the performance of super-spreader identification for a broad range of real-world networks. While doing so, we discover a pattern in the centrality measures involved in the aggregation with respect to the topological structures of the networks used in the experiments. Further, we study the eigenvalues of the Laplacian matrix, also known as Laplacian spectrum, and by using the Earth Mover’s distance as a metric for the spectrum, we are able to identify four clusters to explain the aggregation results.

The super-spreaders are the nodes in a network that can maximize their impacts on other nodes, as in the case of information spreading or virus propagation. The identification of these nodes is very important in many real-world domains, ranging from innovation diffusion^1^, viral marketing^2^, to epidemic disease identification and control^3^,^4^,^5^. Many methods, in particular centrality measure based methods, have been proposed in the past that aim to model and identify the most influential spreaders in complex networks. Among them, K-shell Decomposition method^6^,^7^,^8^ and Expected Force method^9^ have shown better performance than others under various epidemiological models. To evaluate the accuracy of the aforementioned measures various techniques has been employed. The most common one makes use of epidemic simulations^10^ from a single seed node, while the average infection size acts as the spreading characterization of the seed. Another possible way of evaluation is based on real-life tracking (e.g., in information spreading^11^) but, unfortunately, this approach is not always applicable due to the lack of data, for example, in transportation and computer networks. On the other hand, there are many networks where it is possible to quantitatively estimate the probability of spreading at the node level (e.g., based on such information as the number of passengers in a flight or number co-authorship papers) and bind it to the strength of connection between the nodes as a weight value associated with the edge^10^. This way allows for modeling and simulating the impacts of a spreader by taking into consideration the probability of node-level spreading. For instance, in the case of modeling information diffusion in a social network, the individual or meta-population level contact information (e.g., the duration or the frequency of the contact) can play an important role; this information will be reflected in the edge weights of the network^12^.

In essence, a centrality measure computes ranking score for the nodes of a network based on their connectivity characteristics. Various centrality measures have been used to predict the epidemic outcomes of the nodes, with the basic assumption that more centrally the nodes are located in the network, the greater spreading power they will have^6^,^13^,^14^,^15^. The well-known centrality measures, and the most frequently used in this field, include: Degree^16^, Strength^17^, Betweenness^16^,^18^, Closeness^16^, Eigenvector^19^, PageRank^20^ and K-shell^6^,^7^,^21^,^22^,^23^. A novel and more effective measure, known as Expected Force^9^, has been recently proposed with a particular aim for classifying nodes based on their influence in the network. This measure calculates the possible clusters generated from a fixed number of transmission events, and using entropy as aggregation of the clusters, it generates a node ranking. However, most of the previously proposed centrality measures take into account only the topological features of the network, without explicitly considering the varying strengths (or weights) of the connection strength, which are essential in modelling network diffusion processes. It has been shown that a natural extension to the weighted case is possible^24^ for all these measures (as detailed in Supplementary Information) except for K-shell where the ranking is computed from recursive pruning based on the node degrees. To address this issue, a weighted k-shell decomposition^25^,^26^ has been proposed, where the pruning is based on the neighbours’ connection strengths.

Another important aspect is concerned with determining the accuracy of the measures, that is to evaluate which measure can be considered as the best super-spreader predictor. In doing so, a susceptible-infected (SI) spreading simulation^10^ is typically run starting from each node in the network, so as to obtain a ground truth of the node spreading power. Thus, the problem is translated into that of evaluating which ranking, as calculated based on respective centrality measures, is better correlated with the one generated from simulations. A commonly used method is based on computing correlations (Pearson, Spearman and Kendall tau^27^). However, when the aim becomes the identification of the most influential node, just the top section of the ranking would be important. Besides, different methods, like imprecision function^6^ and recognition rate^11^, have also been proposed to evaluate a prefixed percentage of the ranking. This allows to have a better comparison because it increases the resolution on the ranking section where the super-spreaders are located. Generally speaking, for many networks, there is no single measure that over performs all the others among different percentages of ranking. For instance, different measures may perform better in different sections of the ranking^15^.

Due to the aforementioned observations, it remains a challenge to find which measure, if any, can better identify the super-spreader nodes. This is especially true if considering more realistic constrains, such as a spreading model based on the interaction strengths and an evaluation targeted to the top section of the ranking. To this extend, one can raise the following two questions: (1) Is it possible to find a measure that gives a *consistent* performance among different parts of the ranking for different kinds of networks? (2) Is it possible to find a *corresponding patten* between the best predictors and the characteristics of networks? To the best of our knowledge, there have been no studies in the literature that explicitly and adequately address these questions. In order to answer the aforementioned two questions, in this paper, we investigate a meta-centrality approach in which an aggregation method is used to combine different centrality measures and hence obtain a more robust performance in the super-spreader identification. Furthermore, we conduct a Laplacian spectrum analysis to characterize the networks and to evaluate their correlations with the results obtained from the aggregated measures. Throughout our evaluations, a set of real-word networks covering a broad range of domains has been used.

## Results

As mentioned above, there is no single centrality measure that consistently performs as the best predictor. This is because different measures have different objectives, and then based on them, they rank nodes in different ways. In an abstract sense, we may view the ranking computed from a certain centrality measure as that produced by an *expert* that evaluates certain features of a network. Thus, if we could aggregate the rankings (opinions) of different experts, we would be able to improve the final ranking results since each of the aggregated rankings brings its own contribution in the identification. A similar approach has also been adopted in other domains such as meta-search in Web engine^28^, biological databases^29^, and recommendation systems^30^. In doing so, it is essential to find the best aggregation of different rankings so as to obtain an improvement in the results. There have been some classical aggregation methods, such as Borda count method^31^,^32^, Kemeny-Young method^33^,^34^, and Median rank method. Some of the methods, for instance Kemeny-Young method, demand heavy computation even with a few (e.g., four) different rankings. In this regard, Borda count method requires relatively lighter computation, as it performs a scanning of all the rankings plus a sort. In addition, it also handles rankings with ties well^35^, which has been a major problem associated with some of the centrality measure rankings as they assign the same values to different nodes. The original Borda count method considers that every ranking has the same importance. A better performance can be achieved by using just a subset of the rankings or by using a weighted version of the Borda count^32^. Normally, this task involves training data to learn the optimal weighting schema. However, since it is not always possible to have training data, as in our case, other unsupervised models^36^ should be considered. In what follows, we first present a novel heuristic method, based on pairwise correlations, for selecting a subset of rankings so as to obtain an improved Borda count aggregation method. Next, we perform a series of experiments on real-network datasets to show the results of our proposed method. Finally, we correlate the topological features of the considered networks with the results as obtained from the improved Borda count aggregation by means of Laplacian spectrum analysis.

### The improved Borda count aggregation method

Generally speaking, Borda count method takes as input a set of ordered lists *T* = {*τ*_1_, …, *τ*_*n*_}, where each list has the same *C* items in different orders. Let us denote 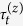 the position of the item *z* in the list *τ*_*t*_. Then, the re-ranking value of an item *z* can be given as follows:


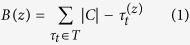


where *B* is a vector with all the ranking items and |*C*| is the cardinality of the item set. Thus, the descending reorder of *B* represents the new aggregated ranking. In this basic form, each rank *τ*_*t*_ ∈ *T* is considered as being equally important, but there could be rankings that have a better accuracy than others. In our present case, the ordered lists correspond to the centrality measure based rankings, whereas the items *C* are the networks’ nodes. As mentioned above, the rankings generated by the centrality measures could be considered as opinions from different experts. Therefore, if a group of experts agree on the same subjects, their opinions could be considered more reliable. In reality, the opinions from experts who do not agree with the trend are still considered reliable, this is because they may still bring in useful alternatives. On the other hand, the situation in which experts are half agreed with each other will be considered unreliable, since their opinions are inconsistent with the mass. Thus, based on this idea of “social” aggregation, we formalize our heuristic method for selecting rankings.The method proceeds in the following steps: *slicing, selection*, and *aggregation*. Let *M* = [*m*_*ij*_]∈[0, 1]^*n*×*m*^ be the correlation matrix, where 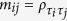 and *ρ* represents the Spearman correlation between the rankings *τ*_*i*_ and *τ*_*j*_. It should be noted that this correlation also enables us to handle any possible disagreement among rankings by assigning it to a negative value. This may happen, for example, when we consider centrality measures such as clustering coefficient^37^,^38^. Indeed, this centrality assigns lower values to more central nodes, and hence it can lead to negative correlation values^39^. However, in the current work, we will not expect to encounter any large disagreement, since our rankings are based on the selection of those centrality measures that are known to be potential super-spreader identifiers, that is, they exhibit similar monotonic trends by their nature. This is also confirmed in all our experiments in that we have obtained only positive values of correlation except one case with a negative value very close to zero. Generally speaking, in handling such cases, we treat negative correlations similar to the uncorrelated ones (i.e., setting their values to zero).

The details of the three steps are as follows:

*Slicing*: This step attempts to select the subsets of ranking that are going to be used later in the aggregation step. To do so, we select a series of subsets for each row *i* in the matrix *M*. Let be 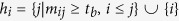 and 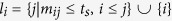, where *t*_*b*_ and *t*_*s*_ are two positive constant thresholds. Thus, we define the following sets:


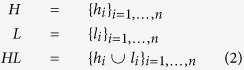


where *H* represents the set containing high correlation subsets, *L* is the one containing low correlation subsets, and *HL* is the one having both. Note that different thresholds can create subsets with different cardinalities; we will further discuss about the threshold selection later. Naturally, one can expect that in this way, there could be many subsets, since each row could have different combinations of correlated rankings.

**Figure 1:**
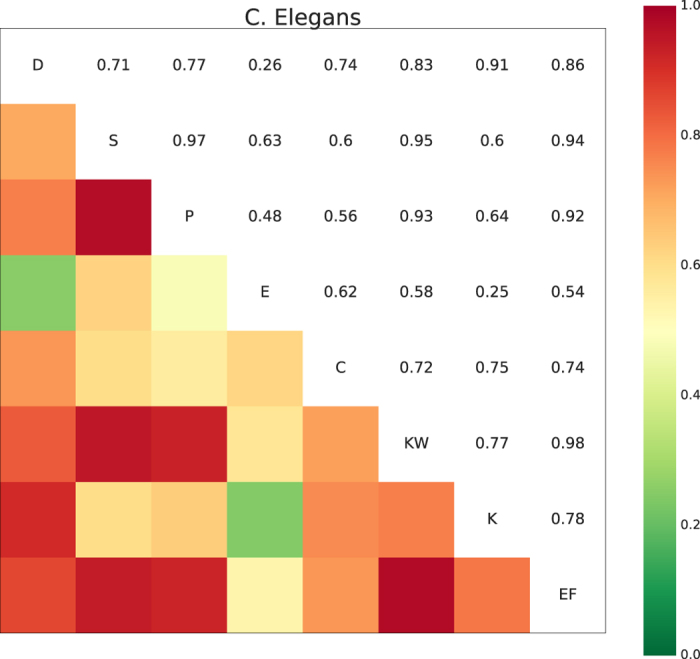
An example of Spearman correlation matrix of C. Elegans network. As a numerical example of the proposed method, we consider just the first row. Thus, we have the sets of *h*_1_ = {1, 6, 7, 8}, *l*_1_ = {1, 4}, and *h*_1_ ∪ *li*  =  {1, 4, 6, 7, 8}, with *t*_*b*_ = 0.8 and *t*_*s*_ = 0.3. Then, *E*(*h*_1_) = 0.460, *E*(*l*_1_) = 0.505, and *E*(*h*_1_ ∪ *li*) = 0.383.

*Selection*: This step is designed to select the subsets of rankings that contain the most informative attributes. We discard some subsets of the ranking based on their entropy values as follows. Specifically, for each subset of the rankings, *X* = *H*∪*L*∪*HL*, we calculate its entropy based on the normalized correlation value. More formally, for each *x*_*i*_ ∈ *X*, where *i* is the index of the selected subset row, we compute the following:
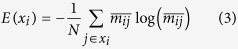
where 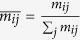, and *N* = |*x*_*i*_| is a scaling factor. This division is used to tune the cardinality of the subsets, in order to pick solutions with a smaller number of rankings. Finally, after the calculation of the entropy, we select the subset 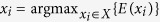 and, to be more reliable, we also keep the subset with the second highest entropy value. Figure 1 presents a numerical example of this step.

*Aggregation*: With the above-mentioned *two* selected subsets, we calculate a new score for each node by performing the Borda count aggregation based on Equation (1). Thereafter, with the descending order of the list, we obtain the new ranking.

### Experiments on real-world networks

In order to evaluate the effectiveness of our proposed Borda count aggregation-based meta-centrality method, we conduct the super-spreader identification on 16 real-world networks from different domains. Table 1 shows the main features of the 16 networks. For each network, we first perform susceptible-infected simulations so as to establish a ground truth ranking for all the nodes in the network for the purpose of evaluation (See Methods). In doing so, we create a descending ranking *I* of nodes in which the first node has the largest infection influence. Next, we compute rankings based on centrality measures and use the results as the input of our aggregation method. The centrality measures used in the experiments consist of: Degree (*C*_*D*_), Strength(*C*_*s*_), Closeness(*C*_*C*_), Eigenvector (*C*_*E*_), PageRank(*C*_*P*_), K-shell (*C*_*K*_), weighted K-shell (*C*_*KW*_), and Expected Force (*C*_*EX*_). It should be noted that here apart from K-shell and Degree centrality measures which are unweighted, all the others are calculated based on the edges’ weights (See Supplementary Information). We keep the two unweighted measures so as to bring in some contributions in the super-spreader identification from the topological perspectives. For consistency of notation, we let C_*_ denote a centrality measure, *S* the node list sorted in a descending according to their ranking scores of C_*_, and *A* the rank generated from the aggregation.

To evaluate the accuracy performances of prediction by the considered centrality measures and by the aggregation, we compare only the top *f* nodes in the ranking generated from the ground-truth simulations and the one generated by a predictor. This evaluation method, also known as recognition rate^11^, can be expressed as follows:


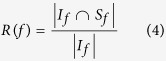


where |*| represents the cardinality of a set, *I*_*f*_ the top section of the ranking generated from simulations, and *S*_*f*_ the top section from a centrality measure. In our experiments, we test six value of *f*, i.e., 0.05, 0.10, 0.15, 0.20, 0.25, and 0.50, and examine the mean and percentile of the recognition rates among them. This allows us to gain a better understanding of the accuracy performance, in particular, concerning the top section of the ranking. For each of the 16 networks, we evaluate all the considered centrality measures along with the two aggregated meta-centrality measures based on the aggregated rankings. Without loss of generality, the parameters of Equation (2) are set as: *t*_*b*_ = 0.8 and *t*_*s*_ = 0.3.

**Figure 2:**
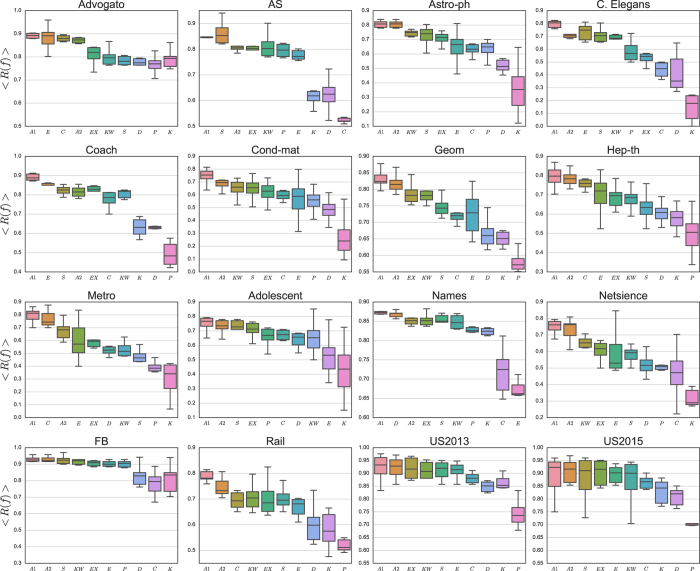
Box-plots showing the recognition rate distribution among different centralities. Each sub-plot shows in a Box-plot the recognition rate variation among *f* values for all the centrality measures and the aggregated ones (as labelled in abbreviation in x-axis). Specifically, the box shows the interquartile range, the segment inside corresponds to the mean value, and two whiskers indicate the maximum and minimum of the range, respectively.

**Figure 3:**
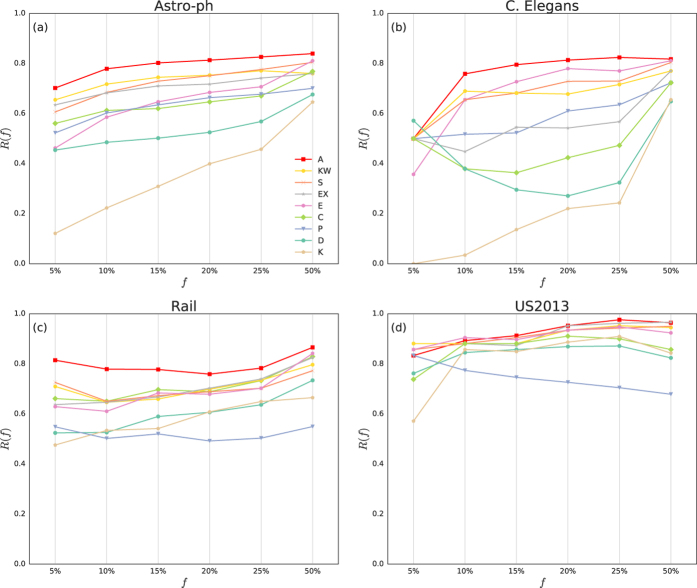
Aggregated solution can better identify super-spreaders as compared to the individual centrality measures. Different values of *f* are given in x-axis, while the corresponding recognition rate changes in y-axis. In this figure, we show the following data-sets: Astro-ph (**a**), C. Elegans (**b**), Rail (**c**), and US2013 (**d**).

The above-mentioned two parameters allow for fine-tuning of our method. Here, we made heuristic choices in setting these values in order to select highly correlated as well as uncorrelated rankings. We conducted a series of experiments with values close to the selected ones, and as a result, we found that these values, as a general setting, could identify the correct centrality measures for the aggregation, consistently among all the networks. In other words, we would not make a customized selection of the parameters specifically for each network. Figure 2 shows the mean and percentile of the recognition rates among the considered *f* range. As shown in Fig. 2, the rankings generated from the aggregation results give the best predictions about super-spreaders for all the networks. In five networks (i.e., C. Elegans, Netsience, Astro-ph, Cond-mat, and Rail), there is a percentage rise of the mean value between 9%–18% as compared to the best single solution. In eight networks (i.e., FB, Geom, Hep-th, Metro, Coach, Advogato, AS, and Adolescent), the percentage rise is between 2–5%. And, in the remaining networks (i.e., US2013, US2015, and Names), the rise is around 1%. Furthermore, in almost all the aggregation results, we observe a decrease in the standard deviation. It should be noted that centrality measures are subject to large variations among different *f*. For instance, Fig. 3 shows the recognition rate values for all the considered centrality measures together with the best aggregation results in four networks. It should also be pointed out that some cases in our experiments, e.g., C. Elegans, Cond-mat, Hep-th, US2013, and US2015, as shown in Fig. 2, where the aggregation selected has the second highest entropy, denoted by *A*_2_ in the subplots, do not lead to the best mean but to a decreased standard deviation value. Therefore, we have used the mean value as the criterion of comparison, and hence the best aggregation from the early-mentioned *selection* step, denoted by *A*_1_ in the subplots, as the proposed solution. For the exact values of the percentages, the best single centrality measure among different *f*, and all the recognition rate plots, check Table S1 and Figures S1-4 in Supplementary Information.

### Network characterization based on Laplacian spectrum analysis

Our next task is to examine whether or not there exists a corresponding pattern (or correlation relationship) between centrality measures used for the aggregation and the networks of certain topologies. To achieve this task, we first characterize each of the networks using its Laplacian spectrum. The Laplacian spectrum analysis has been broadly used^40^,^41^ to describe the topology of the network, as it is capable of providing a model of the global topological features of a network, with no explicit reference to its individual nodes. Specifically, in this paper, we consider a Normalized Laplacian Matrix^42^, which is defined as 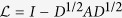, where *A* is the adjacency matrix of a network, *D* is the diagonal matrix of node weighted degrees, and *I* is the identity matrix. The eigenvalues of 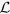 are in the range of 0 ≤ *λ*_1_ ≤ … ≤*λ*_*n*_ ≤ 2, where *n* is the number of nodes in the network^42^. Thus, the Laplacian spectrum of a network refers to the eigenvalue distribution of its Normalized Laplacian Matrix. The Laplacian spectrum characterization provides the information about non-trivial characteristics of a network. There are three well-known characteristics^40^: small eigenvalues imply the presence of community structures^43^,^44^, large eigenvalues reflect the level of bipartiteness^45^, and a concentration of eigenvalues near to one indicates the presence of motifs^41^. At the same time, such a characterization allows for comparisons among networks with different sizes and network topologies. For a pair of networks, it is possible to define and compute a pseudo distance between their Laplacian spectra^46^ (See Methods) and hence to quantitatively determine their similarity. In our work, having defined a distance between two Laplacian spectra, we use a hierarchy clustering algorithm to group the networks into certain clusters depending on their similarities. This cluster analysis proceeds by considering each observation as a single cluster, and iteratively merging two clusters into a larger one based on a linkage criteria. In our current analysis, we have adopted a complete linkage criteria, which means using the couple with a maximum distance as the distance between two clusters.

**Figure 4:**
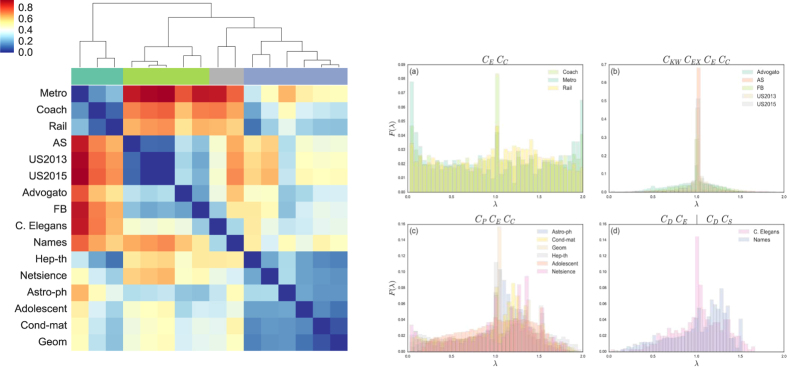
Clusters-map and corresponding spectrum plots. The sub-figure on the left hand side shows the reordered heat map of the spectrum pair distance matrix, with annex dendrogram. The plots on the right hand side present the corresponding histogram plots of the Laplacian spectrum, with eigenvalues in x-axis and their frequencies in y-axis. The four sub-plots correspond to the four identified clusters of the networks, respectively. Note that for the ease of comparison, we have normalized the values of the pair matrix. The title of each sub-plot indicates the set of centrality measures used for the aggregation.

Specifically, for each network, we calculate the eigenvalues of its Laplacian matrix. Then, by using the aforementioned methods, we identify four distinct classes of networks. Each identified cluster exhibits certain special network characteristics that influence the identification of super-spreaders. The first cluster that we have identified, i.e., the first sub-plot shown in Fig. 4a, may be referred to as the cluster of transportation networks, consisting of: Coach, Metro, and Rail networks. In this cluster, each of their histograms is almost symmetric, with a concentration of values in the two extremes. This means a high level of bipartiteness^45^. The aggregation in this network cluster uses *C*_*E*_ and *C*_*C*_ as its main components. This centrality couple are also present in other two clusters; indeed all of them have got some large eigenvalues. The second cluster that we have found, i.e., the second sub-plot in Fig. 4b, is composed of networks with the main part of their eigenvalues close to 1. These networks are: Advogato, AS, FB, US2013, and US2015 networks. The Laplacian spectrum of this cluster is typically developed from repeated additions and duplications of nodes and motifs^41^. In this case, two additional components are involved in the aggregation, namely, *C*_*KW*_ and *C*_*EX*_. The networks in the third cluster, i.e., the third sub-plot in Fig. 4c, include Astro-ph, Cond-mat, Geom, Hep-th, Adolescent, and Netscience networks. Their eigenvalues tend to concentrate between 1 and 1.5. Similarly to the second cluster, the Laplacian spectra of these networks are also developed from recursive additions of triangle motifs^47^. The aggregation in this network cluster uses a new component *C*_*P*_. Finally, the forth cluster, shown as the fourth sub-plot in Fig. 4d, is made of two networks, C.Elegans and Names. Although they do not completely share the same spectrum, they are classified together because they do not have significantly large eigenvalues as in the cases of the other network clusters. In this cluster, the component *C*_*D*_ is the common centrality.

In conclusion, we used the Laplacian spectrum to qualitatively characterize the aggregation results. As a result, for each of the four identified network clusters, we revealed a consistent pattern for the clustered network cases. That is, for each network that belongs to the same cluster, the same set of centrality measures in the aggregation could be found. It would be desirable to make an even stronger conclusion on the connection between network topologies and centralities (or an aggregation of them) that optimally identifies super-spreaders. However, due to the lack of sufficient network samples, it remains to be a future task to quantitatively establish and/or analyze such a connection.

## Discussion

Our work has shown that the aggregation of individual centrality measures results in an excellent predictor for super-spreaders, since it brings in contributions from each of individual ones so as to improve the identification accuracy. Most of the considered centrality measures work in a linear time, but not all. In fact, the closeness centrality does not work well for large networks. To overcome this problem, approximated version of this centrality has been proposed and can be used^48^.

The next important issue to highlight is about the simulation parameterization. Since our aim is to identify the most influential spreaders, the parameter *α* has been set equal to one, in order to perform a simulation that reflects the importance of the weights in the infection propagation. Indeed, if *α* is set to a large value, most of the nodes result in having similar outcomes, and thus we will not have a sufficient resolution to distinguish the importance of the nodes. Therefore, we have decided to fix the parameter *α* based on the following two considerations: First, we would like to bind the infection propagation only to the edge weights so as to reflect as much as possible the real importance of each node. Second, after a series of experiments, in each network, we have noticed that using different values of *α* would only increase the spreading power of every single node, while not affecting the final comparison of the nodes. Indeed, the model^49^ that we have implemented is made to bind the infection propagation to the edge weights (See Methods). Different methods have been proposed^49^,^50^,^51^ to achieve this task, but they were tailor made for particular applications and not suited to the problem of super-spreader identification.

Last but not the least, it should be pointed out that in our current work, we have chosen the SI model as the main spreading model. This is due to the consideration that it fits well with the task of super-spreader identification, in which infected nodes can spread freely until the entire network is fully covered. Nevertheless, it would also be desirable and interesting to examine the results of this work by adopting another commonly used model, i.e., the Susceptible-Infected-Recovered (SIR) model. When using the SIR model, the average numbers of Recovered nodes^6^ at the end of the epidemic spreading could be used for the ground truth ranking. In this study, in order to make sure that our method can also be applied to such a model, we have conducted several experiments by using our aggregation model on SIR simulations. The aggregated solution still gives the best results among all the networks. Interested readers are referred to more details in the Supplementary Information.

## Methods

In what follow, *G*(*V, E*) denotes an undirected, connected weighted network, where *V* represents the set of nodes, and *E* = *V* × *V* the set of edges, and *w*_*ij*_ represents the weight of the edge *e*(*v*_*i*_, *v*_*j*_). Let us denote with *A* and *W* the adjacency matrices of the network *G*, where *A*_*ij*_ = 1 represents the edge *e*(*v*_*i*_, *v*_*j*_) and *W*_*ij*_ = *w*_*ij*_ represents the weight of the connection.

### Spreading models and comparisons

As a measure of spreading power of a node, without loss of generality, here we describe a susceptible-infected (SI) simulation model. In the SI model, at the beginning, all the nodes are in the susceptible state (S) except the one that is in the infected state (I). At each time step of the simulation, the infected nodes will spread the infection to their neighbors, depending on the weights of the connecting edges. The simulation stops when all the network nodes are covered (labeled as I). Different models have been proposed to characterize an infection propagation process based on the connections’ strength^49^,^50^,^51^; in this paper, we adopt the most commonly used model^49^. In this model, the probability that a node *i* becomes infected at the time *t* is given by 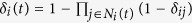, where 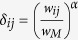, *α* is a positive constant that describes the infection power and allows to tune the power and the speed of the infection, *N*_*i*_(*t*) are the infected neighbors at the time *t*, and *w*_*M*_ is the largest value of *w*_*ij*_ in the network.

To characterize the spreading power of each node, we run a batch of 100 simulations for each seed. In doing so, we record the infection ratio at each time step of a single simulation run in a set, until it has covered all the network nodes (note that the size of the set may vary among different simulation runs). Then, for each generated set, we take the average of its recorded values, and place it in a list *L*_*sim*_ (each value corresponds to a single simulation run). Finally, we calculate the average of the list *L*_*sim*_ previously generated, so as to obtain a single index that represents the spreading power of a node (more details can be found in Supplementary Information). Another way to characterize the spreading power of a node is to consider the average time of full coverage among different simulations. This method has been frequently used^9^, however with the above discussed method we can achieve a better characterization, without losing track of the average time of full coverage. A further discussion can be found in Supplementary Information under the SI evaluation section.

### Laplacian spectrum

The normalized Laplacian matrix of a weighted network *G* is defined as 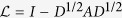, where *A* is the adjacency matrix, *D* is the diagonal matrix of node weighted degrees, and *I* the is the identity matrix. Therefore, for each node *u, v* ∈ *V*, we have:


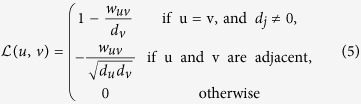


where 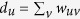. The eigenvalues of 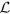 define the spectrum of G, and they are in the range of 0 ≤ *λ*_1_ ≤ … ≤ *λ*_*n*_ ≤ 2, where *n* = |*V*|^42^. For each network *N*_*i*_, we compute the corresponding spectrum of the Laplacian matrix 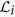 and we define the corresponding eigenvalue histogram as 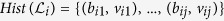, where *b*_*ij*_ represents the *j*−*th* bin and *v*_*ij*_ its frequency. In our analysis, we fix the bin number to 200, such that every bin has a length of 0.01. Since every histogram has got the same number of bins, we can introduce a metric to formally quantify the distance between two spectra. Different metrics have been proposed to achieve this task, either by using a Gaussian kernel estimation^52^ or based on an Euclidean distance between the entire spectrum^44^. Instead, in this paper, we use the Earth mover’s distance (EMD), that is equal to Wasserstein distance, as the metric. This distance falls into the category of cross-bin measures as used to calculate distances between histograms. Computing EMD for multidimensional histograms generally requires the introduction of an optimization framework^53^. Since here we deal with a mono-dimensional histogram, the EMD becomes the distance between the cumulative histograms. More formally, the distance between two spectra 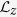 and 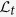 is defined as: 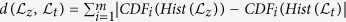, where *CDF*_*i*_ is the cumulative histogram defined as 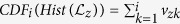. We choose this approach because it is simple and straightforward, and has been proven to be a pseudo metric as well as a graph spectrum distance^46^.

### Data Sets

In this work, all the networks are considered undirected, weighted, and connected. A brief description of the networks used is given as follows: (1) Names (nouns of the King James bible and their occurrences)^54^, (2) C. Elegans (weighted network representing a neural network)^55^, (3) Netscience (co-authorship network of scientists working on network theory and experiments)^56^ (4) FB (online community of students at University of California)^57^, (5) Advogato (online community for developers)^54^, (6) Adolescent (created from a survey that took place in 1994/1995, friendship choices by students)^54^, (7) Geom (authors collaboration network in computational geometry)^58^, (8) Astro-ph (co-authorships between scientists posting preprints on the Astrophysics E-Print Archive)^59^, (9) Hep-th (co-authorships between scientists posting preprints on the High-Energy Theory E-Print Archive)^59^, (10) Cond-mat (co-authorships between scientists posting preprints on the Condensed Matter E-Print Archive)^59^, (11) and (12) US air passengers 2013 and 2015^60^, (13) AS (snapshot of Autonomous System network, where the weights representing the visit-counts in the period of time)^61^, and (14), (15) and (16) Metro, Coach and Rail respectively (network map of UK transportation system, where the edge weights are the average minutes of travel)^62^,^63^.

## Additional Information

**How to cite this article**: Madotto, A and Liu, J. Super-Spreader Identification Using Meta-Centrality. *Sci. Rep.* **6**, 38994; doi: 10.1038/srep38994 (2016).

**Publisher's note:** Springer Nature remains neutral with regard to jurisdictional claims in published maps and institutional affiliations.

## Supplementary Material

Supplementary Information

## Figures and Tables

**Table 1:** Characteristics of the networks used in the experiments.

Network	V	E	<*k*>	D	*λ*_*n*_
Names	1707	9059	10.6	8	1.5000
C. Elegans	297	2148	14.5	5	1.6458
Netsience	379	914	4.8	17	1.7973
FB	1893	13835	14.6	8	1.8667
Advogato	5042	41791	16.6	9	1.8688
Adolescent	2539	10455	8.2	10	1.8696
Geom	3621	9461	5.2	14	1.9513
Astro-ph	14845	119652	16.1	14	1.9550
Hep-th	5818	13644	4.7	19	1.9719
Cond-mat	13861	44619	6.4	18	1.9756
US2013	840	8994	21.4	10	1.9790
US2015	894	8466	18.9	9	1.9899
AS	25241	70669	5.6	10	1.9913
Metro	307	373	2.4	55	1.9941
Rail	2490	4387	3.5	47	1.9971
Coach	1603	2474	3.1	116	1.9987

*V* is the number of nodes, *E* is the number of edges, <*k*> is the average degree of the nodes, *D* is the network diameter, and *λ*_*n*_ is the largest eigenvalue in the Laplacian matrix.
