# Supplementary material for: Super-Spreader Identification Using Meta-Centrality
Source: Sci Rep. 2016 Dec 23;6:38994. doi: 10.1038/srep38994 (PMC5180094; doi:10.1038/srep38994)
Supplement: Supplementary Information [file srep38994-s1.pdf]

Supplementary Information: Super-Spreader Identification Using Meta-Centrality

Andrea Madotto and Jiming Liu

Department of Computer Science, Hong Kong Baptist University, Kowloon Tong, Hong Kong

jiming@comp.hkbu.edu.hk

Contents

1 Terminologies and definitions **2**

1.1 Centrality measures 2

1.2 Spearman correlation 3

1.3 Simulation ranking details 3

1.4 SI evaluation 4

1.5 SIR evaluation 8

2 Computational tools **8**

3 Detailed results **8**

1 Terminologies and definitions

In what follows, $G(V, E)$ denotes an undirected, connected weighted network, where V represents the set of nodes, and $E = V \times V$ the set of edges, and w_{ij} represents the weight of the edge $e(v_i, v_j)$. Let us denote with A and W the adjacency matrices of the network G, where $A_{ij} = 1$ represents the edge $e(v_i, v_j)$ and $W_{ij} = w_{ij}$ represents the weight of the connection.

1.1 Centrality measures

Degree and Strength The Degree centrality is defined as the number of incident neighbors of a node, thus $C_D(i) = \sum_{j=1}^{|V|} a_{ij}$ represents the degree of a node i. A straightforward extension to the weighted case, also called strength [1], is given by $C_S(i) = \sum_{j=1}^{|V|} a_{ij} w_{ij}$, that is the weighted sum of the edge labels. 78

Betweenness and Closeness Betweenness and Closeness centralities make use of the shortest path. The first one calculates the information flow, as the shortest path between each pair, that passes through a node. Thus, the betweenness of node i is defined as $C_B(i) = \sum_{s \neq t} \sigma_{st}(i)/\sigma_{st}$ where $\sigma_{st}(i)$ is the number of the shortest paths between s and t that pass through i, and σ_{st} is the total number of the shortest paths between s and t. The closeness of node i is defined as the reciprocal sum of the distance between i and all the nodes in the network. Formally, $C_C(i) = [\sum_z d(i, z)]^{-1}$ where $d(i, z)$ represents the shortest path distance between i and z. A natural representation of both measures in their weighted versions is obtained using the weighted shortest paths, therefore C_B^w and C_C^w denote the weighted betweenness and closeness, respectively.

Eigenvector and PageRank Eigenvector centrality and PageRank use the neighbour scores to calculate the importance of a node. The first one for each node i is defined as $C_E(i) = \lambda^{-1} \sum_j A_{ij} C_E(j)$. In a more formal way, the eigenvector centrality is the solution of the equation $Ae = \lambda e$, where e is an eigenvector of the adjacency matrix A, and λ is a positive eigenvalue (the existence is guaranteed by the PerronFrobenius theorem[2]). PageRank centrality has been used by Google to rank web pages in its search engine. The original design was for a direct graph. The PageRank of node i is defined as $C_P(i) = \frac{1-d}{|V|} + d \sum_j \frac{a_{ij} C_P(j)}{deg(j)}$, where d is a damping factor (conventionally fixed to 0.85) and $deg(j)$ is the degree of node j. In both measures, the weighted versions, C_E^w and C_P^w, are obtained by using the adjacency matrix A^w. For completeness, the two centralities are calculated using the power iteration method[3].

K-shell K-shell method is based on recursive pruning. The algorithm starts with the pruning of all the nodes with degree $k = 1$. After this first pruning, if there could be some nodes that still have a degree equal to one, then the pruning process continues until there are no nodes with degree one. All the removed nodes are considered as $1 - shell$ and labeled as $K_s = 1$. This pruning and labelling procedure is repeated for the nodes with degree $K \geq 2$ until all the nodes are assigned to the respective shell.

The weighted version[4] does not only consider the degree as pruning rules but also for each node i assign the value of the connections strength between its neighbours. For instance, $k'_i = \sqrt{k_i \sum_j w_{ij}}$, where k_i is the degree of i and $\sum_j w_{ij}$ is the sum of all its incident links. To follow the previous notation, C_K represents the convention K-shell and C_{KW} its weighted version.

1.2 Spearman correlation

Spearman rank-order correlation is a non-parametric measure to quantify the correlation between two rankings. Let τ_k and τ_t be two rankings of a set C with $|C| = n$, and $\tau_k^{(i)}$ the position of the item i in the rank τ_k (the same for τ_t). The Spearman correlation ρ is defined as:

$$\rho_{\tau_k,\tau_t} = 1 - \frac{6\sum_{i=1}^{n} d_i^2}{n(n^2-1)}$$

where $d_i = \tau_k^{(i)} - \tau_t^{(i)}$. The correlation value is equal to 1 indicating that the two rankings have a perfect monotonic relation. The value equal to 0 implies no correlation. Note that when there are rankings with ties, as it sometimes happens in our case, this formula is valid with an average of the tie values[5].

1.3 Simulation ranking details

Algorithm 1 presents the pseudo code of the procedure that assigns a spreading power value to each node trough the Susceptible-Infected (SI) simulation. In the algorithm, AVG($\bar{x}$) is the average value of the vector $\bar{x}$, and SIM(G) represents a single SI simulation run on the network G. The output of the latter is a vector containing the ratios of infected nodes in the network at each time step of the simulation run. The length of these vectors may vary among the 100 runs, since each simulation stops when all the network nodes are infected. This method is chosen in view that there could exist some variations among different simulation runs starting with the same infected node.

Algorithm 1: SI simulation ranking

Data: $G = (V, E)$

Result: A list R of nodes, with the spreading value

```
Initialize R as an empty vector
for v ∈ V do
    Set v as the infected seed
    val ← [ ] /* init an empty vector*/
    for i ∈ [1, 100] do
        sim ← [ ]
        sim ← SIM(G)
        val[i] ← AVG(sim)
    end
    R[v] ← AVG(val)
    Set all the nodes as susceptible
end
```

1.4 SI evaluation

Instead of using the average time of infecting the whole network to measure the spreading power of each node, in our current study, we have adopted a different measurement so as to incorporate more information about the process of spreading propagation. Specifically, we use the average number of infected nodes among the simulations to capture the spreading dynamics. The new measurement is strongly correlated with the average time of full network infection coverage, but at the same time, can also reflect the speed of the infection propagation. The measurement allows for a more even distribution of the values, and thus it is a better characterization of the spreading power.

In the literature, various epidemic models have been used to tackle the problem of super-spreader identification. Two of the most commonly used ones are: Susceptible-Infected (SI) and Susceptible-Infected-Recovered (SIR). In our current study, we have mainly focused on the SI model in our experimental studies, as we believe it allows to adequately characterize the nature of network-based disease propagation. In order to evaluate whether or not the proposed measurement can reflect the average time of full network infection and improve the nodes' spreading power representation, we use all the networks from our data-sets. We run 100 realization of Susceptible-Infected (SI) simulations starting from each node, and we record the average time of full network infection. To simplify our notation, we call the average number of infected nodes (i.e., our measurement) as AVG_I, and the average time of full network infection as AVG_T. Moreover, we compare the results obtained from the two measurements using Sprearman correlation coefficient. In all networks, we are able to find a very high correlation value, except for Adolescent network where the correlation is slight lower. The exact correlation values can be found in Figs 1-4, where we show binned scatter plots between AVG_I and AVG_T. Furthermore, we also show the standard deviation (std) values of the two measurements. We can note that the std values are high in

all networks, and they have similar values in the Astro-ph, AS, and Metro networks. More detailed results are given in Table 1 and Figs 5-8 of Section 3.

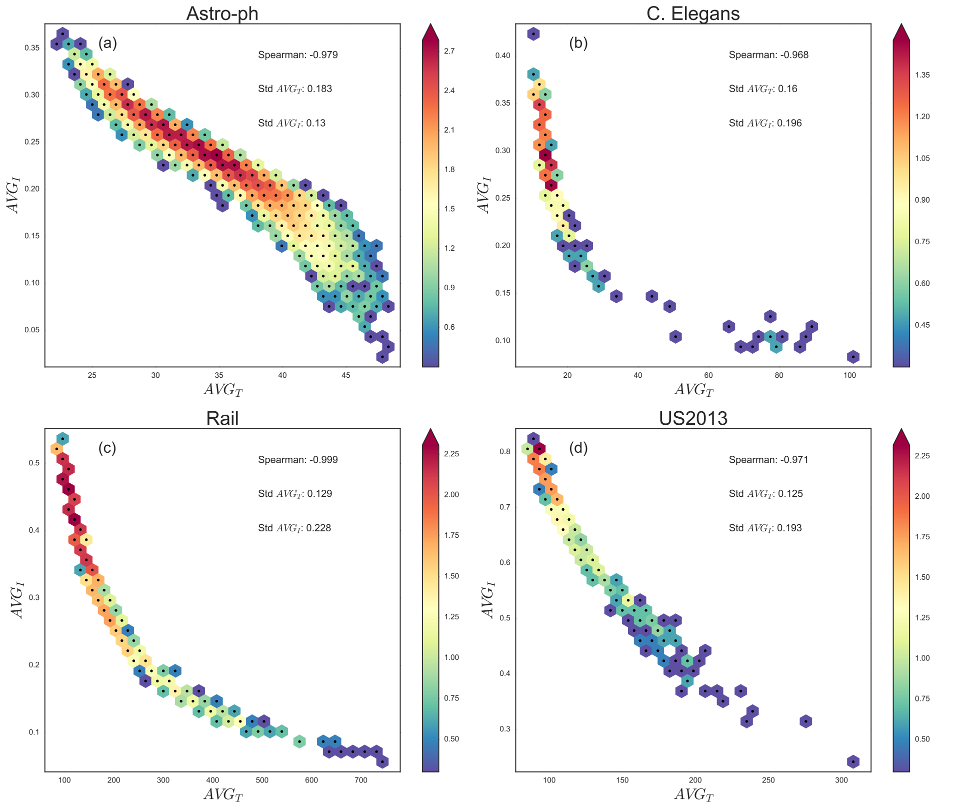

Figure 1: Scatter plots between the average number of infected nodes (our measurement) and the average time of full network infection. Each sub-plot shows the Spearman correlation and the standard deviation of the two measurements. In this figure, we show the following data-sets: Astro-ph (a), C. Elegans (b), Rail (c), and US2013 (d).

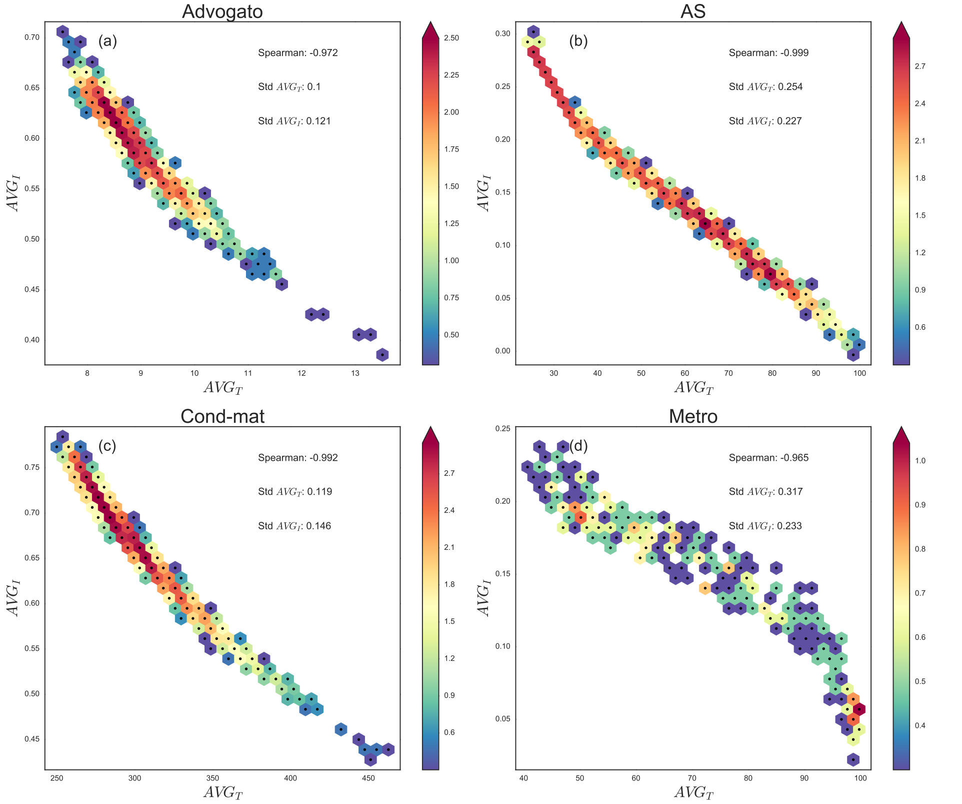

Figure 2: Scatter plots between the average number of infected nodes (our measurement) and the average time of full network infection. Each sub-plot shows the Spearman correlation and the standard deviation of the two measurements. In this figure, we show the following data-sets: Advogato (a), AS (b), Cond-mat (c), and Metro (d).

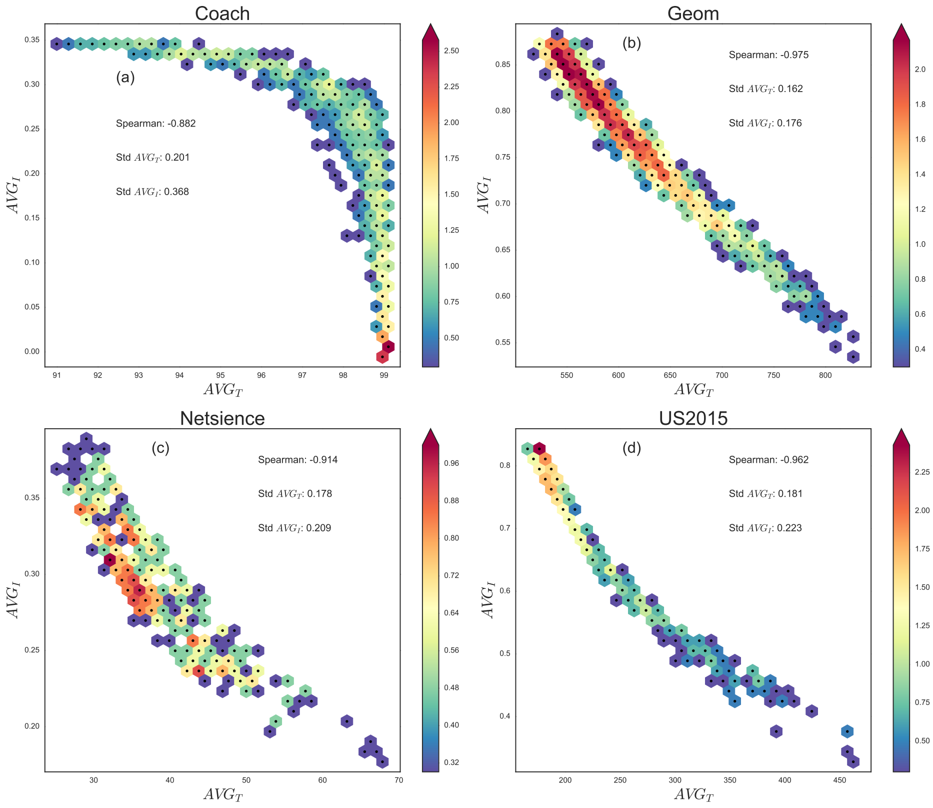

Figure 3: Scatter plots between the average number of infected nodes (our measurement) and the average time of full network infection. Each sub-plot shows the Spearman correlation and the standard deviation of the two measurements. In this figure, we show the following data-sets: Coach (a), Geom (b), Nescience (c), and US2015 (d).

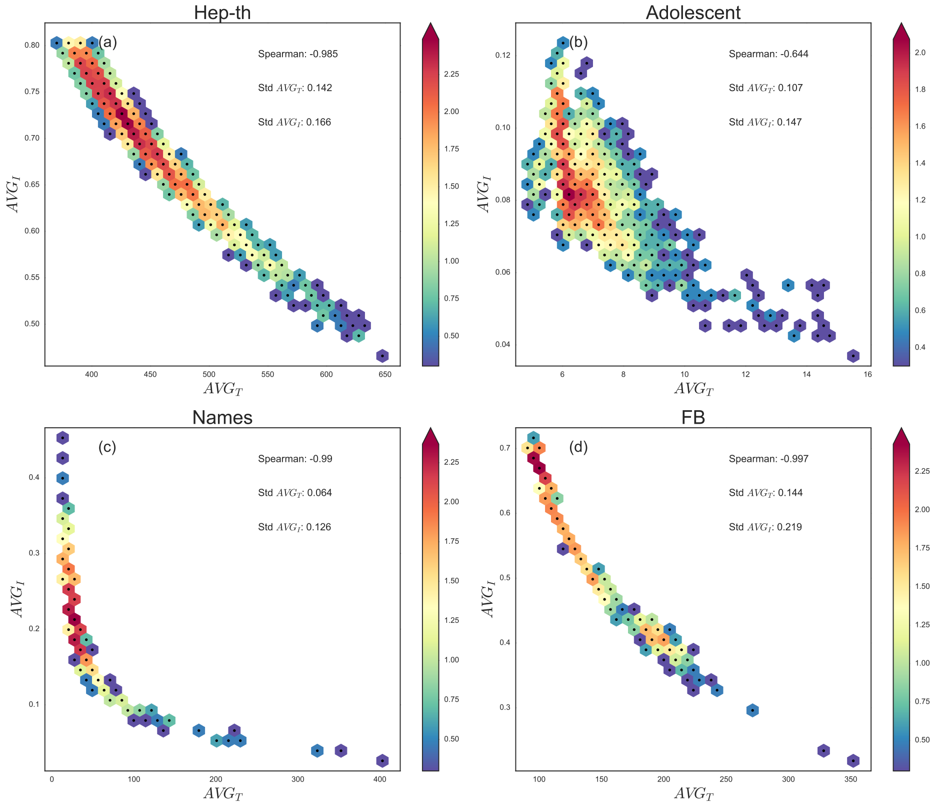

Figure 4: Scatter plots between the average number of infected nodes (our measurement) and the average time of full network infection. Each sub-plot shows the Spearman correlation and the standard deviation of the two measurements. In this figure, we show the following data-sets: Hep-th (a), Adolescent (b), Names (c), and FB (d).

1.5 SIR evaluation

In the preceding section, we have presented the results of our evaluation based on the SI epidemic model. Nevertheless, it is also desirable and interesting to evaluate and show our method using the Susceptible-Infected-Recovered (SIR) model where another state (i.e., Recovered) is added to represent nodes that will not spread the infection anymore. In the case of the SIR model, we can characterize the spreading power of a node by averaging the number of Recovered nodes [6] at the end of the epidemic spreading.

In order to evaluate the generality of our method, we test it in all the networks of our data-sets. We run 100 SIR simulations starting from each node. When a node spreads the infection to its neighbors, it will change its state to Recovered. We record the average number of Recovered nodes at the end of each simulation. Using this as the ground truth ranking for the nodes in the network, we test our method. The aggregated results are found to have the overall best predictions about super-spreaders for the tested networks. More detailed results are given in Table 2 and Figs 9-12 of Section 3.

2 Computational tools

For the centrality measures calculation and all the simulations, we used NetworkX[7], a Python library for network manipulation, except for Expected Force where there was an R code provided by the author. To calculate the Spearman correlation, we used the built-in function of Scipy[8] library that handles rankings with ties. For the calculation of the eigenvalues of the Laplace Matrix, we used the sparse matrix class of the Scipy library[8, 9]. As for visualization, we used matplotlib[10] in combination with the seaborn[11] library.

3 Detailed results

In what follows, we present detailed results of the proposed solutions. In Tables 1 and 2, we show the best singular centrality measures among different values of f and the average mean improvements using the aggregated solutions, while using both SI and SIR models, respectively.
In the figures that immediately follow each of the tables, we display different values of f in the x-axes to show how the recognition factor (y-axes) changes; each figure shows four networks used in our experiments. The last figure in this section shows the reordered heat map of the spectrum pair distance matrix (i.e., spectrum plots) and the histogram plots of the Laplacian spectrum, with eigenvalues in x-axis and their frequencies in y-axis (i.e., cluster-map).

Table 1: The best singular centrality measure among different values of f and average mean improvements using the aggregated solution, using SI as the spreading model. The last column shows the improvements in the standard deviation; the values with minus are the ones where the single solutions have obtained lower standard deviations.

	**5%**	**10%**	**15%**	**20%**	**25%**	**50%**	Δ_{mean}	Δ_{std}
Names	D	EX	D	D	D	D	1.2%	-35.13%
C. Elegans	D	KW	E	E	E	E	10.30%	34.38%
Netsience	D	KW	KW	KW	E	E	19.83%	111.03%
FB	KW	S	KW	S	S	KW	1.26%	19.11%
Advogato	C	C	E	E	E	E	1.97%	15.68%
Adolescent	EX	S	S	S	S	S	3.93%	24.66%
Geom	KW	KW	KW	EX	EX	EX	5.64%	17.26%
Astro-ph	KW	KW	KW	KW	S	E	8.19%	-13.94%
Hep-th	C	C	C	C	C	C	3.66%	-23.71%
Cond-mat	C	KW	S	KW	KW	E	14.95%	19.55%
US2013	KW	E	S	EX	EX	EX	0.53%	-14.94%
US2015	S	C	E	S	EX	S	0.93%	11.03%
AS	E	S	S	S	S	S	1.02%	512.84%
Metro	C	C	C	C	C	C	3.52%	17.14%
Rail	S	S	C	EX	EX	E	12.18%	69.39%
Coach	E	E	S	E	E	E	3.90%	36.31%

Table 2: The best singular centrality measure among different values of f and average mean improvements using the aggregated solution, using SIR as the spreading model. The last column shows the improvements in the standard deviation; the values with minus are the ones where the single solutions have obtained lower standard deviations.

	**5%**	**10%**	**15%**	**20%**	**25%**	**50%**	Δ_{mean}	Δ_{std}
Names	E	KW	E	E	E	E	0.81%	3.03%
C. Elegans	D	KW	S	S	E	E	4.72%	-63.72%
Netsience	S	KW	KW	KW	KW	C	5.83%	-45.31%
FB	KW	KW	S	S	S	S	1.51%	60.56%
Advogato	P	S	S	S	S	S	0.12%	7.63%
Adolescent	EX	P	S	S	S	P	1.51%	13.22%
Geom	KW	E	E	E	E	EX	1.06%	-4.42%
Astro-ph	KW	KW	KW	KW	KW	S	5.98%	-11.78%
Hep-th	S	S	KW	S	S	S	0.64%	5.18%
Cond-mat	S	S	S	S	S	S	0.85%	-0.16%
US2013	KW	S	S	S	S	E	0.04%	-23.28%
US2015	S	S	S	S	E	S	1.03%	-30.76%
AS	S	S	S	S	S	S	0.04%	-14.76%
Metro	S	S	EX	S	EX	EX	4.39%	36.17%
Rail	S	C	E	E	E	KW	6.59%	126.42%
Coach	S	E	E	E	E	S	3.73%	46.86%

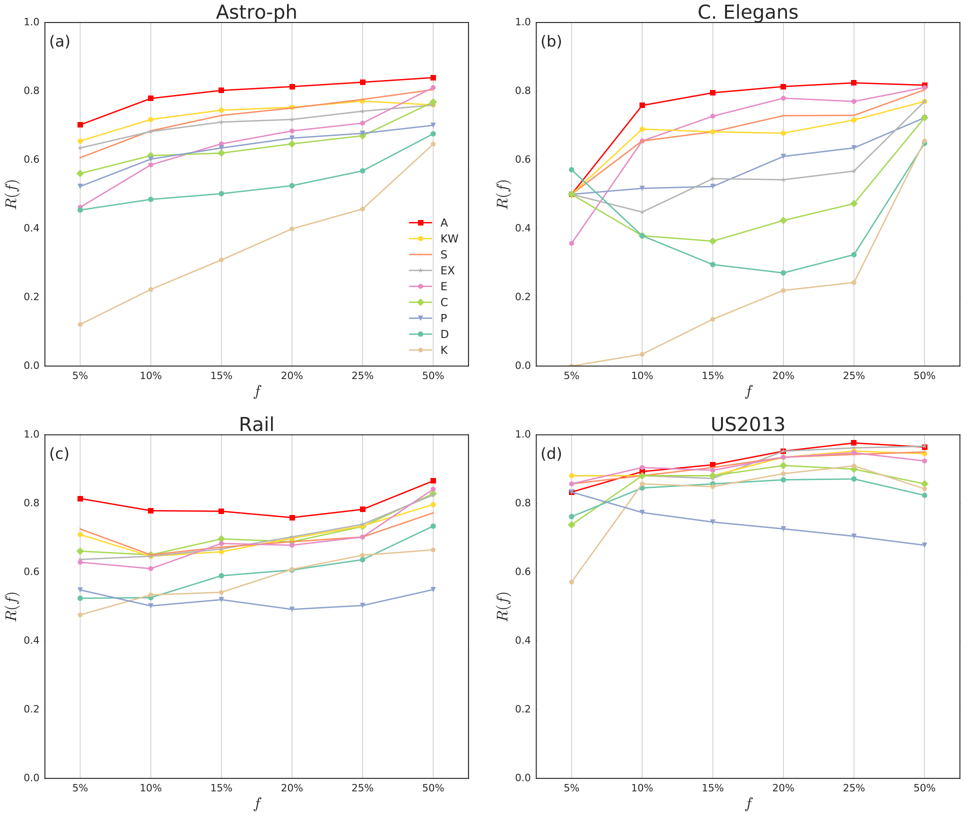

Figure 5: Different values of f in the x-axes to show how the recognition factor (y-axes) changes. In this figure, we show the following data-sets: Astro-ph (a), C. Elegans (b), Rail (c), and US2013 (d).

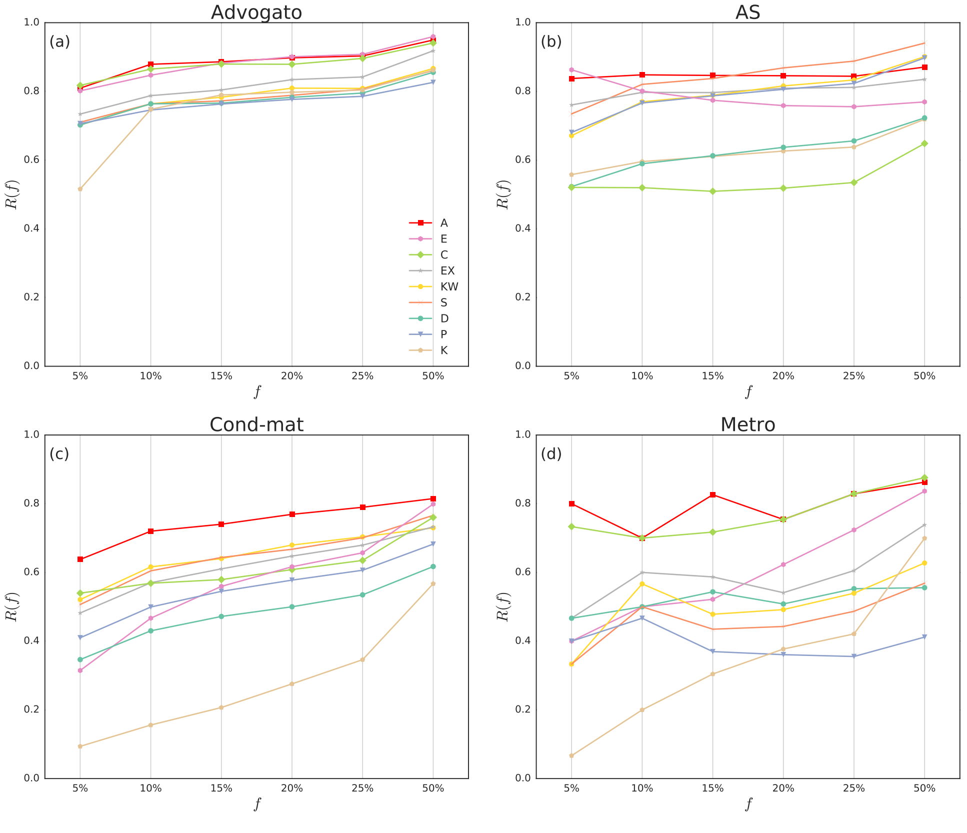

Figure 6: Different values of f in the x-axes to show how the recognition factor (y-axes) changes. In this figure, we show the following data-sets: Advogato (a), AS (b), Cond-mat (c), and Metro (d).

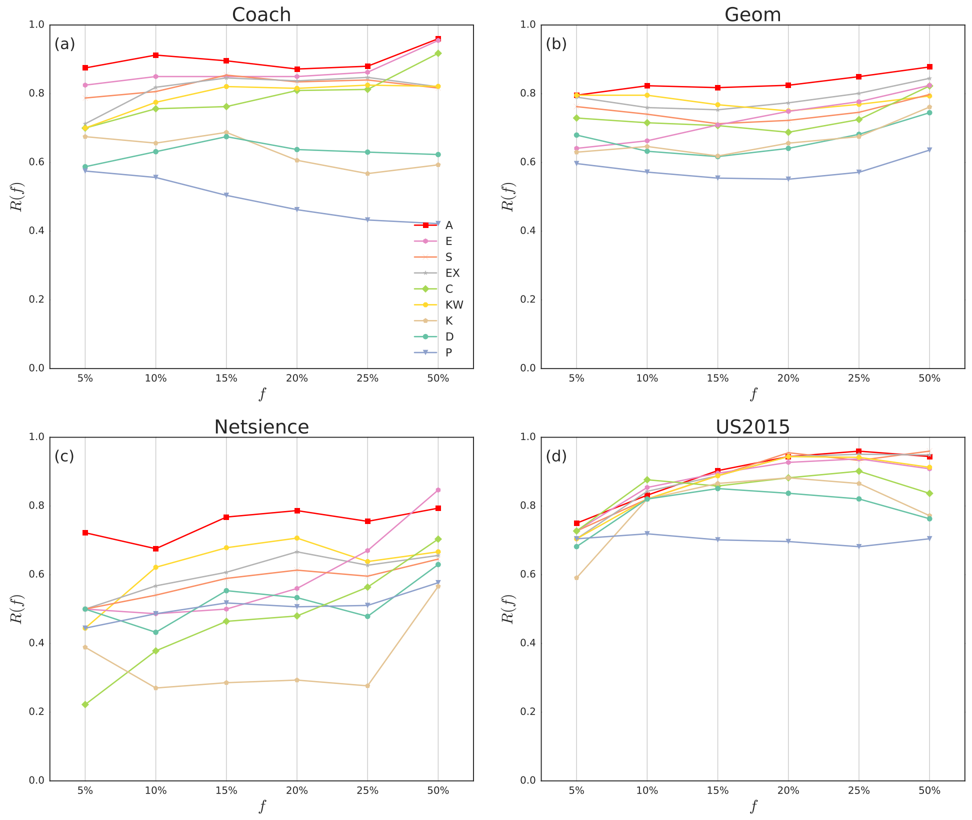

Figure 7: Different values of f in the x-axes to show how the recognition factor (y-axes) changes. In this figure, we show the following data-sets: Coach (a), Geom (b), Nescience (c), and US2015 (d).

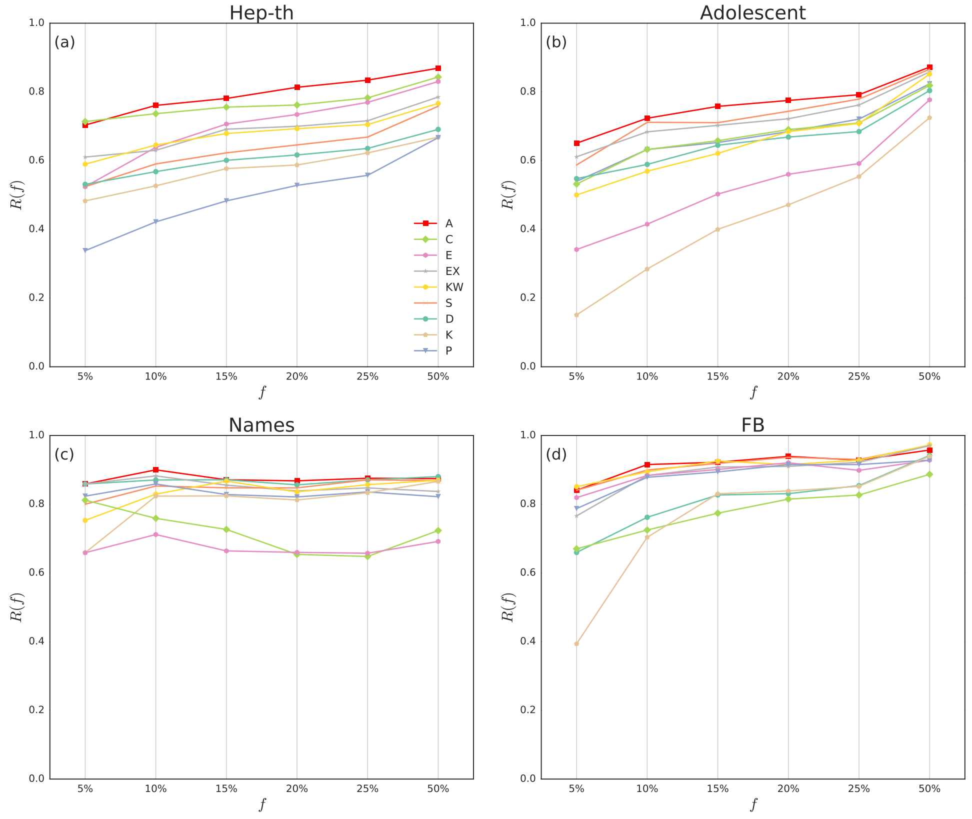

Figure 8: Different values of f in the x-axes to show how the recognition factor (y-axes) changes. In this figure, we show the following data-sets: Hep-th (a), Adolescent (b), Names (c), and FB (d).

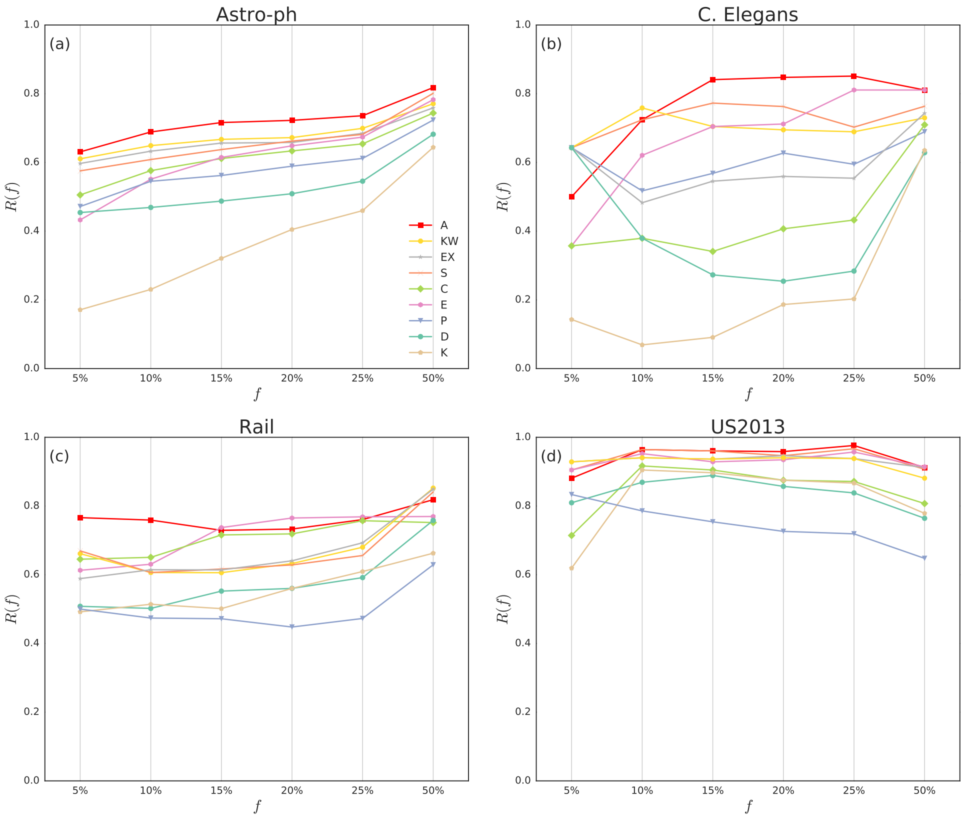

Figure 9: Different values of f in the x-axes to show how the recognition factor (y-axes) changes, using SIR as spreading model. The following data-sets are evaluated: Astro-ph (a), C. Elegans (b), Rail (c), and US2013 (d).

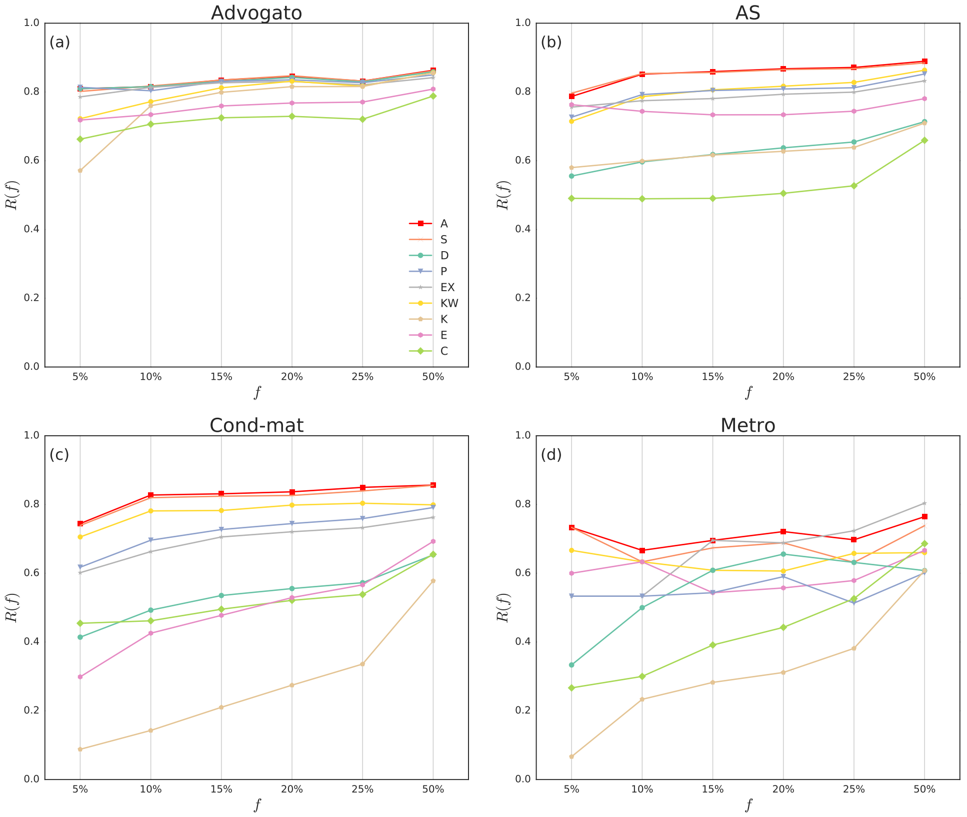

Figure 10: Different values of f in the x-axes to show how the recognition factor (y-axes) changes, using SIR as spreading model. The following data-sets are evaluated: Advogato (a), AS (b), Cond-mat (c), and Metro (d).

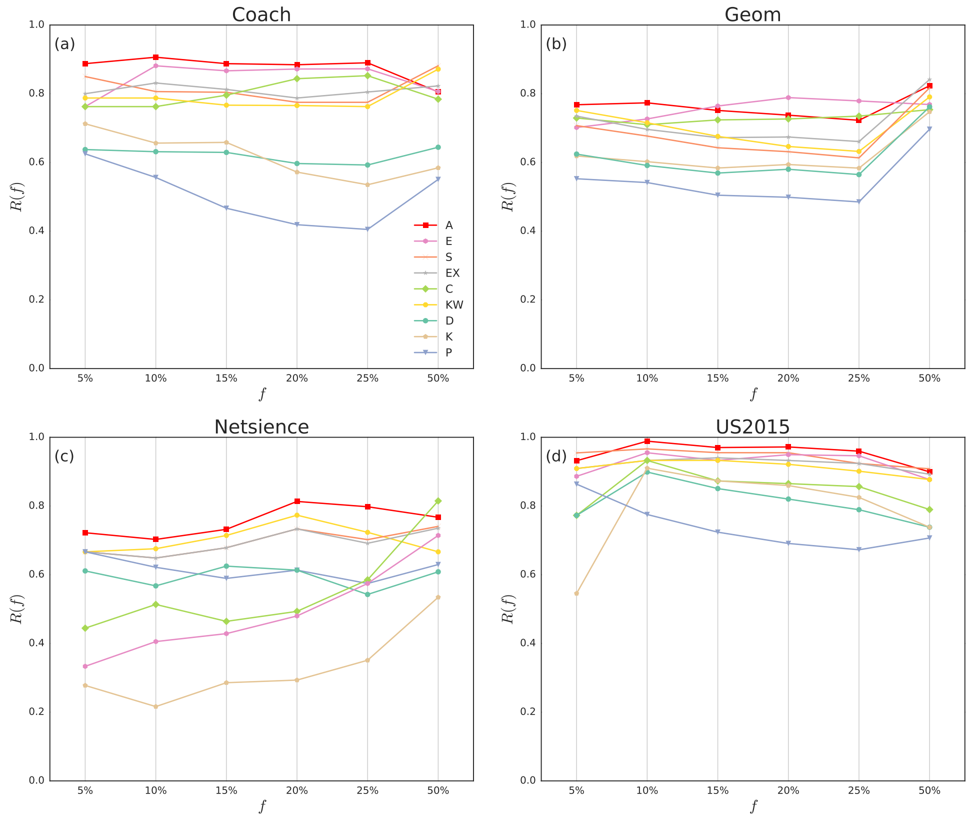

Figure 11: Different values of f in the x-axes to show how the recognition factor (y-axes) changes, using SIR as spreading model. The following data-sets are evaluated: Coach (a), Geom (b), Nescience (c), and US2015 (d).

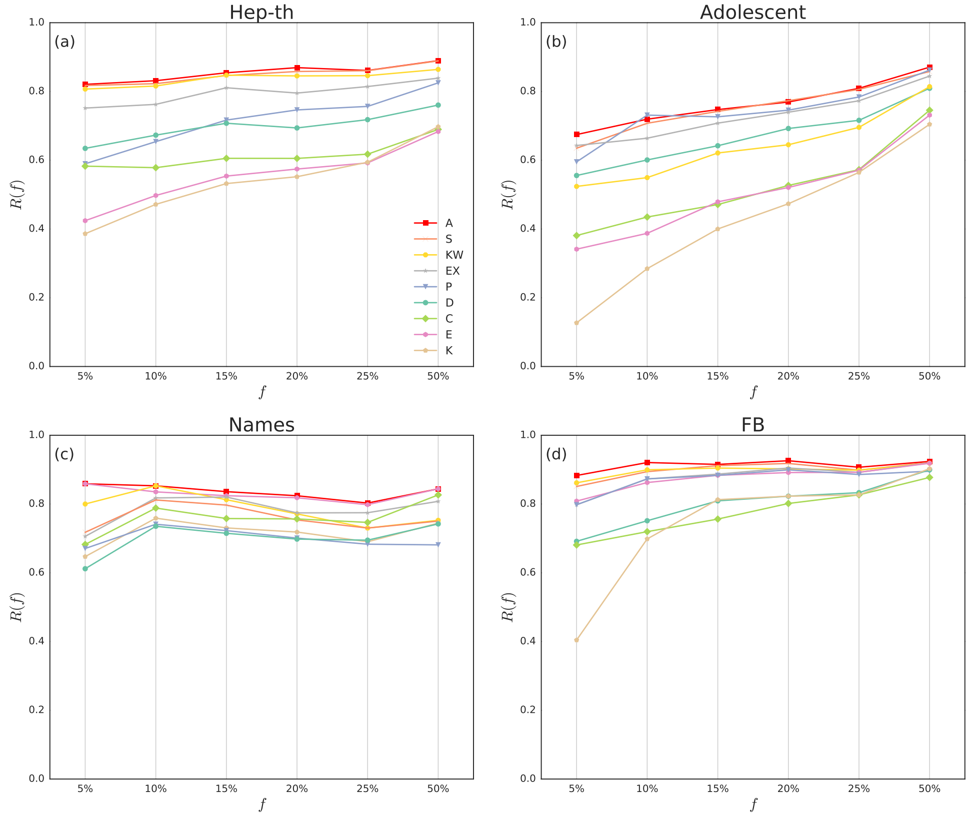

Figure 12: Different values of f in the x-axes to show how the recognition factor (y-axes) changes, using SIR as spreading model. The following data-sets are evaluated: Hep-th (a), Adolescent (b), Names (c), and FB (d).

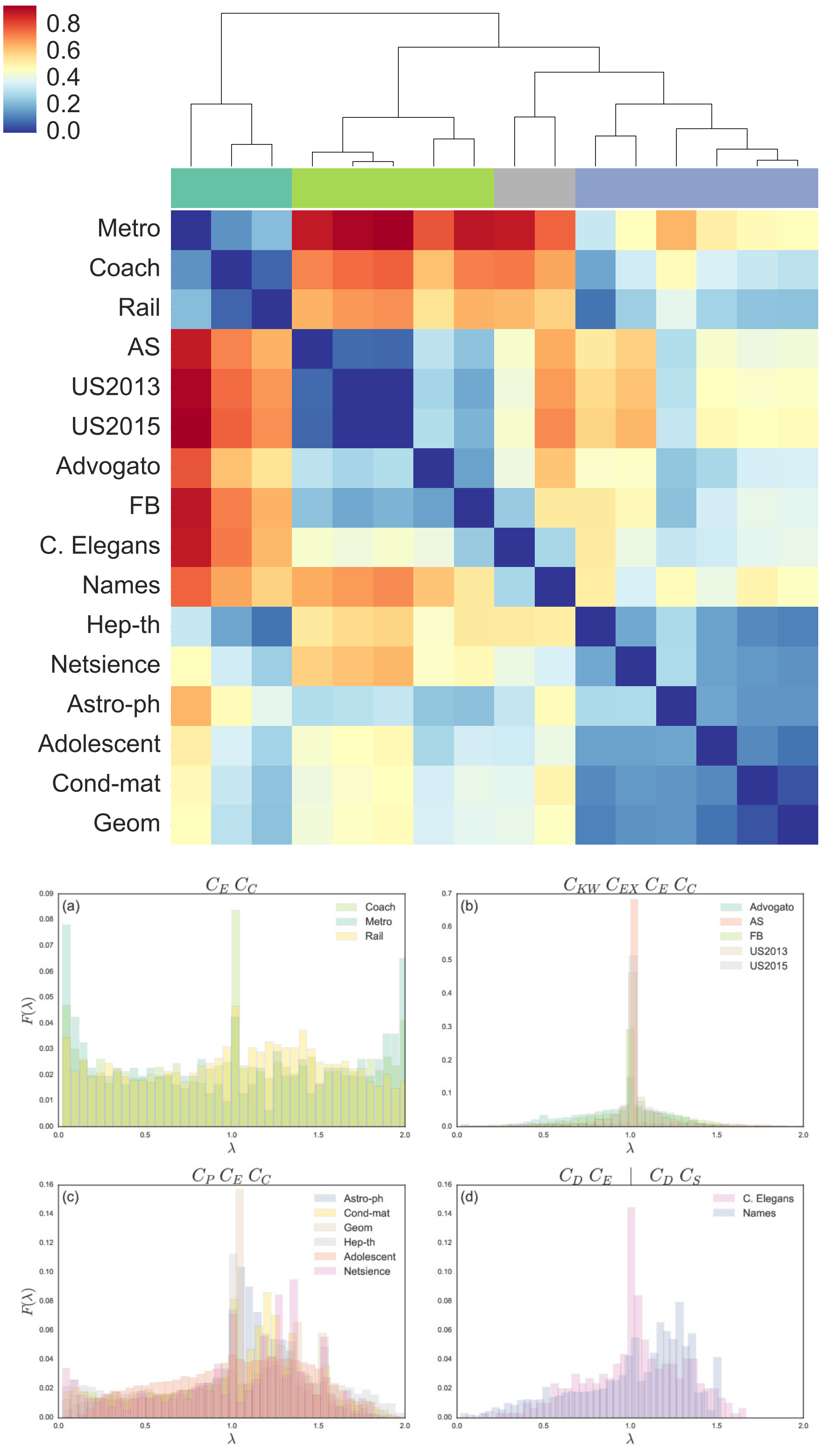

Figure 13: **Spectrum plots and cluster-maps**

References

1. Barthélemy, M., Barrat, A., Pastor-Satorras, R. & Vespignani, A. Characterization and modeling of weighted networks. *Physica A: Statistical Mechanics and its Applications* **346** (2005).
2. Meyer, C. D. *Matrix Analysis and Applied Linear Algebra* (SIAM, 2000).
3. Langville, A. N. & Meyer, C. D. A survey of eigenvector methods for web information retrieval. *SIAM Review* **47** (2005).
4. Garas, A., Schweitzer, F. & Havlin, S. A k-shell decomposition method for weighted networks. *New Journal of Physics* **14** (2012).
5. Taylor, W. L. Correcting the average rank correlation coefficient for ties in rankings. *Journal of the American Statistical Association* **59** (1964).
6. Kitsak, M. *et al.* Identification of influential spreaders in complex networks. *Nature Physics* **6** (2010).
7. Schult, D. A. & Swart, P. Exploring network structure, dynamics, and function using NetworkX. *Proceedings of the 7th Python in Science Conferences (SciPy 2008)* (2008).
8. Jones, E., Oliphant, T., Peterson, P., *et al. Open Source Scientific Tools for Python* (2001).
9. Lehoucq, R., Sorensen, D. & Yang, C. ARPACK Users' Guide: Solution of Large Scale Eigenvalue Problems with Implicitly Restarted Arnoldi Methods. *Software Environment Tools* **6** (1997).
10. Hunter, J. D. Matplotlib: A 2D graphics environment. *Computing in Science and Engineering* **9** (2007).
11. Waskom, M. *et al. Seaborn: v0.7.0* (2016). doi:10.5281/zenodo.45133.
